# Implications of Anaphylaxis Following mRNA-LNP Vaccines: It Is Urgent to Eliminate PEG and Find Alternatives

**DOI:** 10.3390/pharmaceutics17060798

**Published:** 2025-06-19

**Authors:** Jinxing Song, Dihan Su, Hongbing Wu, Jeremy Guo

**Affiliations:** WuXi Biologics, 190 Hedan Road, Waigaoqiao Free Trade Zone, Shanghai 200131, China; song.jinxing@wuxibiologics.com (J.S.); su_dihan@wuxibiologics.com (D.S.)

**Keywords:** LNP, anti-PEG antibodies, immunogenicity, PEG alternatives

## Abstract

The mRNA vaccine has protected humans from the Coronavirus disease 2019 (COVID-19) and has taken the lead in reversing the epidemic efficiently. However, the Centre of Disease Control (CDC) reported and raised the alarm of allergic or acute inflammatory adverse reactions after vaccination with mRNA-LNP vaccines. Meanwhile, the US Food and Drug Administration (FDA) has added four black-box warnings in the instructions for mRNA-LNP vaccines. Numerous studies have proven that the observance of side effects after vaccination is indeed positively correlated to the level of anti-PEG antibodies (IgM or IgG), which are enhanced by PEGylated preparations like LNP vaccine and environmental exposure. After literature research and review in the past two decades, it was found that the many clinical trial failures (BIND-014, RB006 fell in phase II) of PEG modified delivery system or PEGylated drug were related to the high expression of anti-PEG IgM and IgG. In the background of shooting multiple mRNA-LNP vaccines in billions of people around the world in the past three years, the level of anti-PEG antibodies in the population may have significantly increased, which brings potential risks for PEG-modified drug development and clinical safety. This review summarizes the experience of using mRNA-LNP vaccines from the mechanism of the anti-PEG antibodies generation, detection methods, clinical failure cases of PEG-containing products, harm analysis of abuse of PEGylation, and alternatives. In light of the increasing prevalence of anti-PEG antibodies in the population and the need to avoid secondary injuries, this review article holds greater significance by offering insights for drug developers. It suggests avoiding the use of PEG excipients when designing PEGylated drugs or PEG-modified nano-formulations and provides references for strategies such as utilizing PEG-free or alternative excipients.

## 1. Introduction

The past five years have witnessed the successful performance of mRNA-LNP (lipid nanoparticles) during the COVID-19 pandemic [1,2,3]. Particularly those developed by Moderna and Pfizer/BioNTech have emerged as a groundbreaking advancement in disease control, simultaneously sparking a surge in global research and development efforts in this arena [4]. The first approval of mRNA-LNP vaccines also attracted more attention for therapeutic mRNA-based drug to treat various diseases [5]. Furthermore, clinical trials are underway for cancer therapy as a phase-I result of the mRNA neoantigen vaccine to treat pancreatic cancer by stimulating T cells. This has shown to induce high-magnitude T cell responses in 50% of the patients [6]. More recently, in May 2024, the Food and Drug Administration approved a third RSV vaccine, mRNA-1345 (mRESVIA^®^, manufactured by Moderna), to prevent RSV-LRTD (Respiratory Syncytial Virus–Lower Respiratory Tract Disease) in adults aged over 60 years old. In mRESVIA^®^, Moderna uses the same lipid nanoparticles as in its COVID-19 vaccine. This is the third RSV vaccine and the first mRNA vaccine for RSV therapeutics. However, the subsequent pediatric RSV vaccine trials of Moderna are currently on hold. As the mechanism is being investigated, the results have also give rise to a more intra-industrial reflection about mRNA vaccines’ safety, either from the cargo or its delivery system-LNP [7,8].

The innovative mRNA vaccine technology mentioned above employs foreign mRNA, encoding a target antigen. This mRNA is introduced into somatic cells to produce the antigen protein, thereby activating a comprehensive immune response. This transformative approach essentially turns the body into a bio-factory for producing therapeutic proteins [9,10]. To address this process smoothly, many strategies are used to improve the cargo’s stability. For example, mRNA structure or sequence can be modified to improve stability or in vivo transfection. Importantly, the crux of mRNA vaccination lies in the efficient release and transcription of the mRNA cargo by its carrier system [9].

LNP, initially designed for small-molecule drugs, have gained prominence in nanomedicine for their biocompatibility, efficient RNA encapsulation, and ease of mass production [11]. A mRNA-LNP system generally consists of cationic/ionizable lipids, helper lipids, cholesterol, PEG-lipids, and target RNAs. Ionizable lipids interact with negatively charged RNAs [12]. Among these, the PEG-lipid can reduce the aggregation of the particles, controlling the particle size with defined diameters and extending shelf-life [13]. However, concerns have risen regarding the use of polyethylene glycol (PEG) in LNPs, particularly its association with anaphylactic reactions and immunogenicity, prompting a reevaluation of its safety, especially in the context of repeated injections for vaccinations and cancer treatments. The potential immunogenicity of PEG-lipids and the generation of anti-PEG antibodies in response to repeated injections have become pressing concerns. There is an increasing amount of studies that reveal that PEG-lipid is associated with anaphylactic reaction and its immunogenicity is refocusing our attention [14,15,16,17,18,19,20].

Polyethylene glycol (PEG) is a polyether product obtained via the polymerization of ethylene glycol and ethylene oxide, and its chemical structure is HO-[CH_2_-CH-O]_n_-H. Its molecular weight is calculated according to the number of ethylene oxide units. Early studies proved the safety of PEG and its inert and biocompatible properties [21]. PEG has been widely used in food, drugs, and daily cosmetics. PEG can also be grafted to nucleus, protein, and small-molecule drugs via covalent bond, which is a process is called PEGylation. Based on their hydrophilic property, PEGylated hydrophobic drugs can form liposomes in water. This is a commonly used and effective approach to increase stability and prolong circulation time in vivo. By changing the structure of PEG, PEGylation can be used for different purposes.

This review calls for an increased scrutiny of the biosafety of PEG and its conjugates, particularly in RNA vaccines and gene therapies. It discusses the mechanisms of PEG immunogenicity, clinical cases, regulatory measures, and detection methods for anti-PEG antibodies. Additionally, promising PEG alternatives and future perspectives are explored. Our aim is to provide a comprehensive overview of PEG immunogenicity in RNA-related drugs, from research to clinical application, highlighting the need for safer and more effective delivery systems in nanomedicine and mRNA vaccination.

## 2. Immunogenicity of PEG

### 2.1. Immunogenicity of PEGylation

For a long time, PEG has been heralded as a stealth polymer with no immunogenicity or antigenicity. It is widely applicated in biomedicine and daily fine chemicals since it was approval by the FDA as a generally non-toxic substance [22,23,24,25]. However, the increasing reports of hypersensitivity cases linked to PEGylation, coupled with the discovery of anti-PEG antibodies (anti-PEG Abs), have casted a shadow over its previously unquestioned safety profile [20,26,27,28,29,30,31,32]. Headache, back pain, tightness in the chest or throat, and breathing difficulties, among others, are common symptoms of hypersensitivity reactions, and even death has occurred in the most severe cases [14,33]. Anti-PEG Abs, including anti-PEG antibody IgG, IgM, and IgE, are common biomarkers of PEG immunogenicity, indicating potential immunogenic responses or the possible connection between PEGylated medicine and hypersensitivity reactions [34]. These antibodies can bind with PEGylated drugs, potentially reducing their half-life, accelerating blood clearance (ABC) of PEGylated drugs, or causing premature drug release. Moreover, the generation of anti-PEG Abs may trigger hypersensitivity reactions, some of which can be life-threatening, through complement activation [35,36]. Notably, the ABC phenomenon and associated hypersensitivity typically manifest after the second injection, a phase known as effectuation. Anti-PEG Abs have been generated during the induction phase after initial dose exposure and without immunogenetic response.

**Figure 1:**
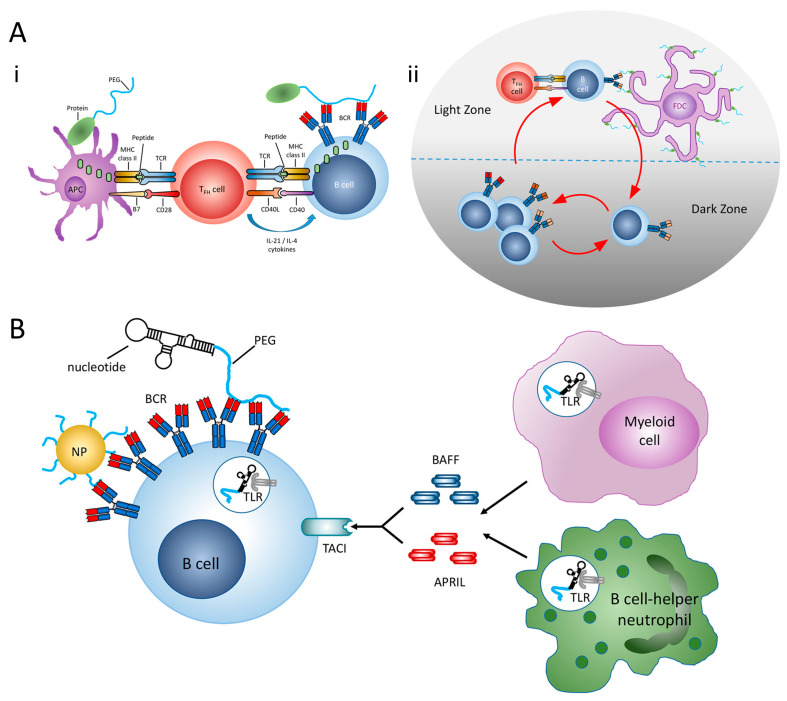
Immune response to PEG. (**A**). Thymus-dependent immune response against PEG. (**i**) Antigen-presenting cells take up pegylated proteins and present digested peptide fragments to activate specific TFH cells (**ii**) Anti-PEG B cells undergo rapid proliferation and somatic hypermutation in the dark zone of a germinal center. (**B**) Thymus-independent immune response against PEG [37]. Copyright © 2021 American Chemical Society.

Anti-PEG Abs responses can be elicited via the classical T-cell-dependent pathway or the T-cell-independent pathway regarding anti-PEG Abs to recognize thymus-dependent (TD) or thymus-independent (TI) antigens, as shown in Figure 1A. In the TD pathway, B cells are activated by the cross-linking of PEG epitopes to B-cell receptors, leading to the differentiation into plasma blasts that secrete anti-PEG IgM Abs (Figure 1B). This response is further amplified with the aid of follicular helper T cells (TFH cells), resulting in a robust anti-PEG Abs response [37]. Conversely, in the TI pathway, PEG polymers directly act as TI antigens, enabling B cells to generate antigens that bind to PEG without T cell involvement. Understanding the immunogenicity of PEG and its mechanism is crucial for safeguarding host cells, as the phagocytic clearance of PEG or PEGylated substances may impact the body’s defense functions. The generation of anti-PEG Abs contributes to hypersensitivity reactions to PEG or PEGylated drugs, with different immune responses producing varying anti-PEG Abs [38,39]. The accumulation of anti-PEG Abs, through complement activation and/or immune cells response, increases the likelihood of hypersensitivity, especially when antibody levels and drug concentrations are high. These reactions also raise concerns about the immunogenic potential of PEG-like polymers used in drugs. Exploring the interactions between polymers and anti-PEG Abs could pave the way for the development of innovative alternatives, mitigating the risks associated with PEGylation in therapeutic applications.

### 2.2. Factors Affecting PEGylation Immunogenicity

As the mechanisms of PEGylation immunogenicity are being investigated, it is also crucial to explore the factors that affect the immunogenic response clinically in order to further address this dilemma. Generally, PEG lipid structural characteristics, grafting density on LNP surface, and the drug types encapsulated in LNP are the main aspects under discussion.

#### 2.2.1. Structure Features

PEG lipid structure features, including chain length (molecular weight, MW), chain structure (linear or branched), terminal groups, and PEG-lipid linker, are discussed in detail.

*Molecular Weight (Chain Length)*. PEG molecular weight can influence the biological performance and delivery efficacy of PEGylated LNP [40]. Longer PEG chains are favored for prolonging the circulation life of drugs and achieving favorable biodistribution in vivo [41]. Generally, PEG lipids with longer chains (eg. ranging from 20 kDa to 60 kDa) are commonly used to modify small molecules, peptides, and oligonucleotides [42,43,44]. Meanwhile, PEGs of 1 kDa to 5 kDa are reported to formulate with LNP or antibodies [45]. PEG2000 formulated with COVID-19 mRNA-LNP vaccines are also reported to trigger hypersensitivity. However, it has been observed that anti-PEG antibodies, particularly IgG and IgM, show a preference for binding to PEG lipids with a molecular weight of 3 kDa to 5 kDa [46,47], potentially increasing the likelihood of an immune response [37]. But PEG with shorter chain length, such as PEG400, PEG600, and PEG800, was also reported to induce a strong ABC effect [48]. As both long and short PEG chains can induce immunogenic responses, PEG2000 is still commonly used in LNP formulations to achieve extended circulation life and a relatively acceptable adverse effect. This suggests a nuanced approach to PEGylation, where a combination of longer and shorter PEG chains could enhance drug safety and circulation while minimizing immune responses [49].

*Chain Type (Linear or Branched)*. In addition to chain length, the structure of PEG chains, linear or branched, also significantly influences performance and efficacy of PEGylated nanoparticles. Zhao et al. investigated the influence of the PEG chain structure on the ABC phenomenon [50]. Nanoemulsions, modified by linear PEG (DSPE-mPEG_40k_) PE_40k_ and branched PEG derivatives (DSPE-mPEG_2,40k_) PE_2,40k_, respectively, were evaluated. PE_2,40k_ did not induce the ABC phenomenon after repeated injections, and it maintained longer circulation times than DSPE-mPEG_2k_ and DSPE-mPEG_40k_. A lower level of anti-PEG IgM secretion may explain the results. The study provided reference for eliminating the ABC phenomenon by modifying PEG lipid chains into a branched structure for novel PEG alternatives. For shorter branched chains, branched (mPEG114)_2_-DSPE (PEG5 kDa) was also reported to exhibit better stealth properties in liposomes with respect to linear ones [51]. Notably, the stealth property of PEG lipid can promisingly reduce the risk of PEGylation immunogenicity, while systemic bioavailability and circulation time should also be considered together.

*Terminal Group*. Studies have shown that in PEGylated liposomes, CH_3_O-PEG induces a higher level of anti-PEG IgM compared to OH-PEG and NH_2_-PEG [31]. These insights offer valuable guidance for designing PEG with lower immunogenicity by modifying end-groups or adjusting the self-assembly process within nanoparticles. PEG, as the hydrophilic site, is usually conjugated with a hydrophobic block, while anti-PEG antibody IgM preferentially binds to the interface between the PEG chain and hydrophobic blocks of PEG-conjugates [52]. These insights offer a valuable guidance for designing PEG with lower immunogenicity by modifying end-groups or adjusting the self-assembly process within the nanoparticles.

*Linker with Lipid*. The PEG lipid used in LNP formulation contains hydrophilic site and a hydrophobic site, which are linked by a short linkage. After degradation of the linkage, the drug is de-PEGylated, which is reported for its attenuated ABC phenomenon [53,54]. In some derivatives, a single ester bond connects the PEG and lipid parts, allowing for easy de-PEGylation through an esterase cleavage of PEG in circulating blood [55]. Although many types of linkage have been used in PEG lipids, such as disulfide [56], phosphoramidate [57], hydrazone [58], vinyl ether [59], and pentanamide [60], more investigation is necessary to figure out the immunogenicity performance of these PEG lipids in LNP.

#### 2.2.2. Grafting Density

**Figure 2:**
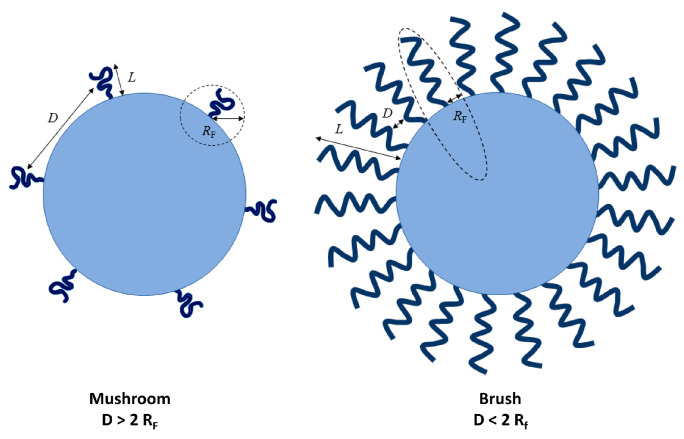
Brush and mushroom conformation of PEG on a nanoparticle surface [61]. *D* represents the distance between the adjacent PEG grafts; *L* represents the thickness of the grafted PEG layer; *R_F_* represents the Flory radius of the PEG graft (the diagrams are drawn not to scale) Copyright © 2020 Nanomaterials.

The density and confirmation of the hydrophilic chain in PEG lipid can affect the stealth property of PEG conjugates anchored on the surface of LNPs. The specific conformation of PEG formed on the liposome surface can be predicted via ratio of the grafting distance (*D*) and the Flory radius of the PEG coils (*R_F_*) as *R_F_/D*, in which *D* is the distance between the two closest PEG grafts and inversely proportional to PEG density on the surface, and *R_F_* is directly dependent on PEG molecular weight and also related with the thickness (*L*) of the graft PEG layer. At low grafting density (*R_F_/D* < 1 or *L = R_F_*), PEG chains adopt a “mushroom” conformation. Conversely, at higher grafting densities, in a “brush” conformation (where *D* < *R_F_* and *L* > *R_F_*), PEG chains are fully extended outward on the surface (Figure 2) [61,62]. This flexibility allows PEG molecules to transition from a “mushroom” to a “brush” conformation as density increases [61]. It has been reported that this transition facilitates a reduction in phagocytic uptake by competing with the adsorption of plasma proteins and decreasing the adsorption of serum albumin [63]. For example, LNP formulated with brush-shaped polymer lipids proved lower immunogenicity than commercially used DMG-PEG lipids. Also, when anti-PEG IgG and IgM levels in mice were tested after seven days of the second injection, the group of brush-shaped PEG lipids exhibited more than 50% higher anti-PEG abs level than the linear PEG group. Accordingly, the brushed PEG lipid LNP showed similar mRNA delivery efficacy after the first and second doses, while the linear PEG LNP presented a c. 87% decrease in mRNA transfection in vivo [64].

#### 2.2.3. Encapsulated Drug

The occurrence of PEG immunogenic response or ABC phenomenon also varies with the type of drug delivered [65]. PEGylated nanocarriers containing cytotoxic drugs are less proponed to trigger the ABC phenomenon. This reduced likelihood may be attributed to the inherent capacity of the drug to inhibit the activity of cells that produce anti-PEG antibodies, such as splenic B cells. For instance, Doxil^®^, a clinically utilized PEGylated liposome encapsulating the cytotoxic drug doxorubicin (DXR), has been reported to not induce the ABC phenomenon [66]. Kiwada et al. revealed that the empty PEGylated liposomes prompted the ABC phenomenon in an inversely dose-dependent manner, where a lower dose resulted in higher complement activation. Meanwhile, repeated injection of liposomal DXR did not elicit complement activation in rat serum [67]. In contrast, non-cytotoxic drugs or immunostimulants (such as plasmid DNA, small interfering RNA, message RNA, etc.) delivered via PEGylated nanocarriers are more likely to provoke PEG immunogenicity. This leads to the generation of anti-PEG IgM antibodies and subsequent rapid clearance, especially after repeat injections [33,68,69]. The dosage of the drug also plays a significant role in the occurrence of immune responses: a proportional increase in the level of IgM was observed with the dose of PEGylated nanoparticles from 0.1 to 20 mg [70], highlighting the complexity of PEG immunogenicity.

#### 2.2.4. Other Factors

It is evident that PEG immunogenicity is not a straightforward issue but, rather, a complex interplay of factors, such as PEG structure, grafting density, chain length, and the type of encapsulated drug. Other factors, like the pre-existed anti-PEG antibody induced by the daily used chemicals, can play a nonnegligible role during the process of PEGylation immunogenicity [71,72,73]. It is reported that anti-PEG Abs was detected in over 40% of the healthy people and higher levels of both anti-PEG IgM and anti-PEG IgG in females than in males [72]. Assessment of pre-existing anti-PEG Abs levels can help mitigate the risks associated with clinical use. Additionally, dosages [19,74,75] (mainly the lipids being administrated) were reported as higher dose of lipids aroused lower ABC. Furthermore, the time intervals between repeated injections [76,77], accordingly, also increased the anti-PEG Abs levels when the time was between 4 and 7 days. In addition, the administration route is also a non-negligible factor that impacts PEGylated LNP-induced ABC [75]. Bolus intravenous administration showed less causative of ABC as the slower rate of exposure for immune response. However, subcutaneous injection tended to enhance ABC [75]. More effective solution is necessary, as subcutaneous injection is the main route to realize the therapeutic effect of mRNA-LNP vaccines. These findings underscore the importance of considering the physical properties of PEG in the design of PEGylated therapeutics, aiming to minimize immunogenicity while maximizing the stealth properties of drug delivery systems. Understanding the interplay between PEG’s physical features and its interaction with anti-PEG antibodies can lead to the development of more effective and less immunogenic drug delivery platforms.

### 2.3. Clinical Immune Responses Triggered by Anti-PEG Ab

Since the first approval of GRIS-PEG^®^ in 1975, the FDA has greenlit nearly 20 PEGylated drugs, with many more undergoing investigations for clinical application. However, an increase in PEGylation-related hypersensitivity reports has emerged. Notably, the Pfizer–BioNTech mRNA COVID-19 vaccines, which contain PEGylated formulation components, have been associated with allergy cases [68,78]. The UK’s initial mass vaccination effort encountered two probable anaphylaxis cases in elderly individuals with known allergies to food and drugs. Following this, the FDA’s emergency use authorization led to the vaccination of nearly 2 million healthcare workers without known allergies, among whom anaphylactic reactions were observed within 10 min post-vaccination. Subsequent reports indicated that the Pfizer COVID-19 mRNA vaccine’s anaphylaxis rate was about ten times higher than that of previous vaccines, approximately 1 in 100,000 compared to 1 in 1,000,000 [79].

Similar PEG-associated anaphylaxis or hypersensitivity has been reported with antibody and small-molecule drugs, such as Jivi^®^ [80], Palynziq^®^ [81], Revolixys^®^ kit [42,71,73,82], and Adynovate^®^ [83,84]. The Revolixys^®^ kit, designed to assess the required degree of anticoagulation reversal to reduce bleeding, encountered allergic-like reactions during its Phase II clinical trial, initially linked to the 40 kDa PEG chain of pegnivacogin. Further research confirmed that pre-existing anti-PEG antibodies could inhibit the anticoagulant aptamer’s activity by binding to PEGylated aptamers, triggering allergic reactions [16].

In cancer therapy, BIND-014, a nanoparticle designed to deliver docetaxel with a hydrophilic PEG targeting the prostate-specific membrane antigen (PSMA), aimed to treat various cancers, including prostate, non-small-cell lung, and advanced cervical, head, and neck cancers. Despite its success in two trials, BIND-014 failed to treat head and neck cancers [16]. Facing significant debt, BIND Therapeutics declared bankruptcy and was acquired by Pfizer for USD 40 million. The failure of BIND-014, alongside the complexity of the tumor microenvironment and tumor heterogeneity, underscores the need to consider PEG’s role in potential off-target effects or premature drug removal due to the interactions between anti-PEG Abs and PEG on the nanoparticle surface [85]. These instances serve as a reminder that the “stealth” polymer PEG may not always equate to “health” [86].

### 2.4. Related Regulatory Measures

Despite PEG being widely utilized in numerous pharmaceuticals and having been classified as a Generally Recognized As Safe (GRAS) material by the FDA since 1973, there has been a growing number of immunogenic cases associated with PEG [32]. Notably, clinical instances of anaphylaxis have been reported in relation to COVID-19 mRNA vaccinations and other PEGylated drugs. In 2021, the FDA and the Emergency Care Research Institute collaborated to assess the safety and compatibility of PEG as a material for medical devices [87]. This partnership involved a thorough literature review to evaluate the current understanding of PEG’s biocompatibility in medical devices, highlighting an escalating concern among regulatory bodies. That same year, the FDA issued a caution against the use of ultrasound contrast agents in patients with known PEG allergies due to the risk of potentially life-threatening type-I immediate hypersensitivity reactions [88]. The period between 2019 and 2021 saw an increase in reported adverse reactions, particularly following the commencement of COVID-19 vaccinations, leading to hypotheses linking PEG sensitization from these vaccinations to adverse reactions observed with ultrasound enhancing agents.

Furthermore, the FDA has documented reports of side effects in the pediatric population, including seizures, tremors, headaches, and sedation associated with long-term PEG use. In 2023, the FDA released guidance on immediate actions concerning the potential public health risks posed by diethylene glycol and ethylene glycol, which may be present as impurities in PEG. Recent research and scientific reports have shed light on the immunogenicity induced by PEGylation, especially given PEG’s crucial role in RNA delivery systems. There is a pressing need for specific regulatory enhancements and improvements to ensure safer utilization of PEG. This endeavor will require comprehensive feedback, increased awareness, and the proposal of potential mitigations, marking a significant journey towards addressing these concerns.

## 3. Anti-PEG Antibodies Detection

The rising instances of immunogenic reactions to PEG may also be linked to advancements in the detection of anti-PEG Abs. Passive hemagglutination represents one of the earliest, most rapid, and convenient methods for identifying anti-PEG Abs. In this technique, PEGylated erythrocytes clump together in the presence of anti-PEG Abs, with the extent of hemagglutination measured as a titer. From this, the approximate concentration of anti-PEG Abs is deduced based on the degree of agglutination. While this method is notably fast and cost-effective, its sensitivity and accuracy leave much to be desired. Consequently, a variety of more sensitive detection techniques have been developed, including Western blot (WB), acoustic membrane microparticle technology (AMMP), enzyme-linked immunosorbent assay (ELISA), flow cytometry, and surface plasmon resonance technology (SPR). The specifics of these methods and their applications are discussed as follows and listed in Table 1.

### 3.1. Western Blot

Western blot (WB) is a widely utilized assay in molecular biology, employed to detect specific proteins or to compare their expression levels across different tissues [89]. This technique models the interaction between antibodies and antigens [90]. Initially, a dye-conjugated PEGylation antigen is incubated with samples to form an anti-PEG Abs complex. This is followed by an enrichment step, where the complex is separated using sodium dodecyl sulfate-polyacrylamide gel electrophoresis. The separated complex is then transferred onto a polyvinylidene difluoride membrane and blocked with 5% bovine serum albumin to prevent non-specific binding. Subsequently, the membrane is incubated with human-positive anti-PEG Abs standards, followed by a washing step to remove unbound antibodies. Detection of the blots is achieved through chemiluminescence, facilitating comparison and analysis [91].

While WB is comprehensive and involves multiple steps, its accuracy may be compromised during the process, and it is limited to detecting only one type of protein at a time. Furthermore, the validation of antibodies is crucial for reproducibility, and a standardized approach for quantifying data is yet to be established. However, fluorescent WB presents a promising alternative for protein detection, as it relies on fluorescence intensity for signal recording. This method offers more precise quantitative results and enables simultaneous analysis of multiple proteins. While this analytical approach remains unexplored in current field applications, its implementation necessitates specialized reagents to address inherent membrane autofluorescence, which is a critical prerequisite for ensuring measurement precision compared to conventional WB protocols. Nevertheless, this methodology demonstrates significant potential as a viable supplementary technique to standard WB assays, particularly in scenarios requiring enhanced signal specificity.

### 3.2. Acoustic Membrane Microparticle Technology

**Figure 3:**
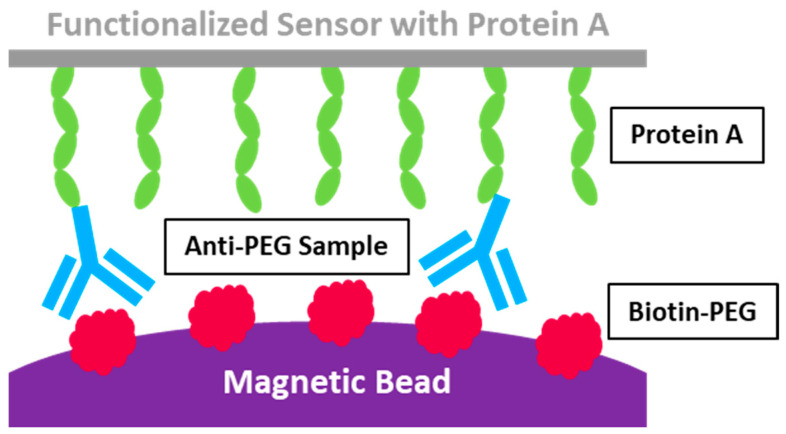
AMMP assay schematic [92]. Copyright © 2015 American Association of Pharmaceutical Scientists.

BioScale’s Acoustic Membrane Microparticle (AMMP) technology innovatively merges magnetic beads, sound wave sensors, and bio-capture protein detection methodologies. This approach employs magnetic beads coated with Protein A to isolate target proteins from biological samples, as shown in Figure 3. It then measures the variations in acoustic frequency resulting from surface quality alterations before and after the acoustic sensor binds with the magnetic beads. This process enables micro-quantification and enhances the sensitivity of detection [92]. In comparison to conventional optical detection technologies, AMMP offers superior detection sensitivity, a broader detection range, and quicker detection speed. This technique is exceptionally adept at identifying proteins in complex, low-abundance samples, such as cell lysates or serum, without being affected by baseline noise interference. It boasts a detection limit as low as 1000 ng/mL for anti-PEG IgG, setting a new standard for sensitivity. However, in the subsequent method validation experiment, this method exhibited poor reproductivity specifically for anti-PEG Abs detection and was deemed unsuitable for clinical sample analysis [93].

However, a notable limitation of AMMP is its specificity in quantifying anti-PEG IgM, primarily due to the exclusive use of Protein A as the protein capture agent. Protein A predominantly binds with IgG, which restricts its ability to accurately quantify other types of antibodies, such as IgM. This specificity underscores the need for further refinement in the technology to broaden its applicability across different antibody types.

### 3.3. Enzyme-Linked Immunosorbent Assay

The enzyme-linked immunosorbent assay (ELISA) stands as the most prevalent technique for detecting anti-PEG Abs, typically conducted in a microtiter 96-well plate coated with PEGylated substances to capture anti-PEG Abs from test samples [94]. Following the attachment of secondary antigens or Abs, the assay is visualized through an enzyme reaction, with results quantified using a microplate reader. The concentration of Abs is determined using a linear function derived from a series of standard dilutions of known concentrations. However, maintaining consistent affinity between testing Abs and standard Abs presents a challenge, leading to results that represent relative, rather than absolute, concentrations of target Abs [95].

The specificity of the PEG coating can significantly affect the binding efficiency with anti-PEG Abs, introducing variability into the results. Moreover, the choice of detergent for washing away nonspecific proteins is a critical variable in the assay and Tween 20 can meet the requirement as a detergent. Meanwhile, Tween 20 is found interfering with the measurement of anti-PEG Abs [72]. However, according to the research by Kozma and colleagues, there is no direct evidence between the slightly higher A450 signal and Tween 20 interfere with the anti-PEG Abs measurement, though the signal, when Tween 20 concentration was at 0.1%, was about 0.2 higher in the A450 signal than the concentration at 0.05% [96]. Meanwhile, 0.05% is a widely used Tween concentration as the detergent for anti-PEG Abs detection [97]. Similarly, washing with 0.05% Triton X-100 can significantly enhance the assay’s sensitivity [94]. Additionally, 3-[(3-cholamidopropyl)-dimethylammonio]-1-propanesulfonate (CHAPS) is reported as the detergent in several anti-PEG Abs ELISA protocols [9,98,99]. Another way to improve the method accuracy involves a pre-treatment, the extraction of the anti-PEG antibodies in human serum via a bead extraction method, followed by further analysis in anti-PEG Abs ELISA [95]. These findings underscore the importance of carefully selecting reagents and pre-treatments prior to the ELISA test to minimize interference and improve the method’s accuracy [96].

### 3.4. Flow Cytometry

In addition to ELISA, flow cytometry has emerged as a highly sensitive assay leveraging fluorescence signals for detection. The typical protocol involves incubating PEGylated polymer beads, often TentaGel^®^-OH nanoparticles, with plasma samples. This is followed by centrifugation to facilitate the binding of IgG or IgM with fluorescent dye-labeled anti-IgG or IgM and the resuspension for flow cytometry analysis [100]. This method capitalizes on the PEG-Ab interaction and allows for the discrimination of Abs isotypes through the distinct fluorescence emitted by anti-IgG or IgM.

Fang et al. utilized flow cytometry to detect anti-PEG IgG and IgM in human blood plasma [91]. They optimized the incubation process by shaking, which facilitated the interaction between TentaGel^®^-OH beads and diluted human plasma. Applying this method, they analyzed 300 healthy plasma samples, finding concentrations of anti-PEG IgG ranging from 39 ng/mL to 18.7 μg/mL and anti-PEG IgM from 26 ng/mL to 11.6 μg/mL. To validate the specificity of anti-PEG Abs, the authors corroborated their findings through Tween-20 interference, preincubation with 20 kDa PEGylated polystyrene beads, and ELISA. The inter-assay and intra-assay variation coefficients were reported as <0.5% and <16%, respectively, showcasing the reliability, accuracy, and robustness of the flow cytometry assay in detecting anti-PEG IgG and IgM Abs in human plasma.

However, it is important to note that the incubation conditions may not fully replicate the binding dynamics of drug-PEG and anti-PEG conjugates in vivo, as the PEGylation process on a polymer matrix differs from the in vivo conditions. Additionally, the lack of a fluorescence standard for this assay presents a challenge for quantification and comparison across studies [101].

### 3.5. Surface Plasmon Resonance Technology

**Figure 4:**
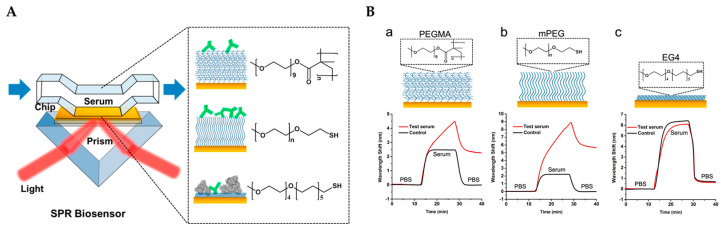
(**A**). Scheme illustrator of the SPR technique of anti-PEG Abs detection. (**B**). The gold chip surfaces were modified by a PEGMA polymer brush (**a**), mPEG (**b**), or EG4 SAM (**c**) and tested by both the negative control (black) and anti-PEG (red) serum samples [102]. Copyright © 2017 American Chemical Society.

Compared with all the methods based on enzyme reactions or fluorescence, surface plasmon resonance technology (SPR) offers an ultra-sensitive alternative, with a detection limit as low as 1 ng/mL anti-PEG Abs. This method enables the monitoring of biomolecule interactions on the surface of SPR sensor chips, allowing for the quantification of absolute anti-PEG Abs concentrations and the discrimination of Abs isotypes. Jiang and his colleges have advanced this field by developing an SPR technique that utilizes sensor chips modified with poly [poly (ethylene glycol) methyl ether methacrylate] (PEGMA), methoxy-PEG (mPEG), and oligo (ethylene glycol) (EG4) (Figure 4) [102]. The PEG coating layer not only minimizes nonspecific protein adsorption but also serves as the antigen for capturing anti-PEG Abs in samples, thus generating signals during SPR analysis.

To reduce the impact of nonspecific proteins, a 5% serum dilution is initially flowed over the sensor chip for 15 min before testing the sample. Following a wash with PBS, the serum sample is introduced to the sensor chips and the resulting wavelength shift signal is recorded. Among the three coating polymers tested, mPEG demonstrated superior performance, exhibiting the most significant wavelength shift (5.6 nm) after a 20-fold dilution of the sample and showcasing its exceptional capability to measure the absolute concentration of anti-PEG Abs. The detection process, completed within 40 min, achieves a detection limit of 10 ng/mL for anti-PEG IgM and 50 ng/mL for anti-PEG IgG, marking this approach as fast, sensitive, and reliable for both academic and clinical applications.

Nevertheless, polymer coating on the sensor chips could influence the detection limit and is similar to the flow cytometry method, while its interaction condition differs from in vivo binding. Thus, it cannot fully replicate in vivo PEG-Abs interactions. Additionally, the requirement for specialized and costly instrumentation for detection is a consideration that cannot be overlooked.

**Table 1:** Anti-PEG Abs detection methods comparation.

Method	Description	Pros	Cons	Detection Limit *	Ref
Western Blot	Main steps: Incubating the PEGylated antigen with samples to form antibody–antigen complex; separating the complex; separating the complex that is transferred to the membrane and blocked to prevent non-specific binding. It is incubated with anti-PEG Abs standards and detected for analysis.	High specificity; relatively lower detection limit	Accuracy compromised during the process; only one type of protein can be detected at once	0.125 μg/mL for anti-PEG IgM 0.750 μg/mL for anti-PEG IgG	[90]
Acoustic Membrane Microparticle Technology	Main steps: Protein A isolation; recording the variations in acoustic frequency resulting from surface quality alterations before and after the acoustic sensor binds with the magnetic beads; calculating the results.	Superior detection sensitivity; broader detection range; quicker detection speed	It showed poor reproductivity for clinical samples.	1000 ng/mL for anti-PEG IgG	[92]
Enzyme-linked Immunosorbent Assay	Main steps: Capturing anti-PEG Abs from test samples in 96 wells coated with PEGylated substances; attaching secondary antigens or Abs; visualizing through an enzyme reaction. The Results are quantified using a microplate reader.	High specificity; relatively lower detection limit; the most widely used for anti-PEG Abs quantitative detection	The results are relative values and vary related to standard curve changes.	100 ng/mL for anti-PEG IgM 1 μg/mL for anti-PEG IgG	[102]
Flow Cytometry	Main steps: PEGylated polymer beads incubated with plasma samples; centrifugation to facilitate the binding of IgG or IgM with fluorescent dye-labeled anti-IgG or IgM; resuspension for flow cytometry analysis.	High sensitivity	Lack of fluorescence standard to compare results across studies.	26 ng/mL for anti-PEG IgM 39 ng/mL for anti-PEG IgG	[91]
Surface Plasmon Resonance Technology	Main steps: PEGylated polymer is coated onto the sensor chips to capture anti-PEG Abs; quantitative analysis is achieved by recording the wavelength shift proportional to the anti-PEG Abs level on the sensor chip.	High sensitivity, quantification of absolute anti-PEG Abs concentrations	A special and expensive instrument is required; polymer coating on the sensor chips could influence the detection sensitivity	10 ng/mL for anti-PEG IgM 50 ng/mL for anti-PEG IgG	[92]

* The detection sensitivity demonstrates significant protocol-dependent variability especially for Western blot and ELISA.

## 4. Solutions to PEGylation Immunogenicity

**Figure 5:**
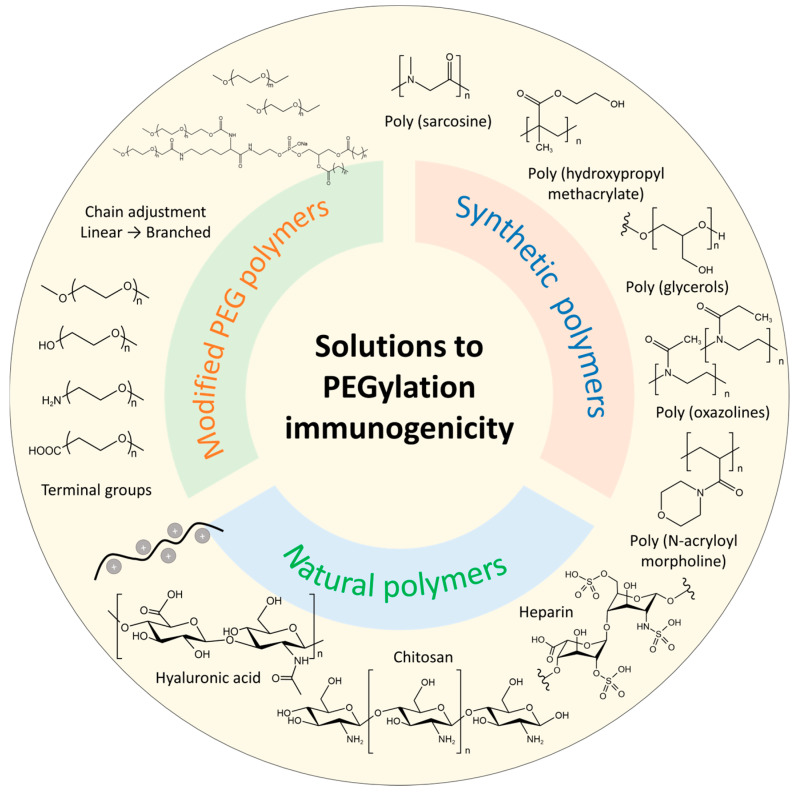
Solutions to PEGylation immunogenicity.

With PEGylation immunogenicity exhibiting more severity, especially in the application of mRNA-LNP COVID-19 vaccines, solutions to reduce or eliminate immune response become increasingly significant and draw tremendous attention. To a certain extent, changing dosage, controlling injection time intervals, and updating drug types could mitigate the ABC phenomenon. Other strategies primarily focus on modifying PEG polymers and exploring alternatives to PEG; these are highlighted as more efficient solutions to this issue in Figure 5 and discussed in detail in the following section.

### 4.1. Modified PEG Polymers

According to the factors affecting PEG immunogenicity, modifying the PEG lipid structure is used to ameliorate ABC and other immunogenicity responses. As different PEG chain lengths, either long or short lengths can cause the ABC effect, and the branched PEG lipids were reported to be an alternative approach [50]. For example, Zhao et al. found that using branched PEG lipid derivatives DSPE-mPEG_2_, n (n = 2, 10, and 20 kDa) to modify nanoemulsions. Furthermore, liposomes did not induce the ABC phenomenon under multiple injections [50]. IgG and IgM are specific to different PEGylation architectures with liposomes or proteins with a single PEG chain, a single branched PEG chain, or multiple PEG chains. The results might be attributed to the fact that branched PEG lipids induced a lower level of anti-PEG IgM. Furthermore, the DSPE-mPEG_2, n_-modified drug also exhibited better in vivo antitumor efficacy [50]. Though related modification has not been used in the RNAs-LNP system, the attempt in LNP is promising to eliminate PEGylation immunogenicity. However, the chain number needs further investigation to equilibrate circulation, immunogenicity, and drug efficacy.

Additionally, changing the terminal group of PEG lipids is another approach to solve this issue. Methoxy PEG is extensively attached to proteins, peptides, liposomes, and drugs to improve stability and efficacy. Meanwhile, PEG terminated with methoxy group was found to dominate the affinities with antibodies compared to hydroxyl group terminal PEG [103,104]. For instance, commercial mRNA-LNP vaccines, including BNT162b and mRNA1273 SARS-CoV-2 mRNA, which use one linear chain of mPEG, increase the levels of anti-PEG Abs [40]. By adjusting the terminal group of PEG lipid, liposomes modified by PEG terminated with hydroxyl group could attenuate or even eliminate the triggering of the ABC phenomenon. However, its safety needs further investigation, especially under multiple injections, as the ABC phenomenon induced by anti-PEG Abs is a complicated process and may require complemental activation to elicit a more severe immune response.

### 4.2. Alternative Polymers

Novel alternative polymer is a better choice to replace PEG from related products. Modifications are far from being satisfying and proper alternative polymers are essential, as the degradation process of PEG may also bring harmful by-products, including reactive oxygen species. This finding also facilitates many industries, not only in the pharmaceutical field but also some cosmetic producers who are trying other polymers to replace PEG. The reported PEG alternatives include synthetic polymers and natural polymers.

#### 4.2.1. Synthetic Polymers

Through polymerization reactions, many PEG-free polymers or their derivatives are reported to delivery RNAs, such as poly (glycerols) (PGs) [105], poly (oxazolines) (POX) [106,107,108], poly (N-acryloyl morpholine) [31], poly (hydroxypropyl methacrylate) (PHPMA), zwitterionic polymers [109,110], and poly (sarcosine) (PSar) [111,112]. Among these polymers, PSar is emerging as a promising alternative to replace PEG in lipid nanoparticles for RNAs delivery. First synthesized by Wesseley et al. about a century ago, PSar is a non-ionic and hydrophilic polypeptoid obtained by sacrosine (a kind of non-coded amino acid) through open-ring polymerization [113]. PSar has drawn increasing attention and has been regarded as a stealth polymer in drug delivery. Initially, PSar showed its potential to design PEG-free protein conjugates [114]. Lu et al. [114]. modified PSar onto interferon-α 2b conjugates with PEG-IFN as control. In vivo results showed that the half inhibition concentration (IC50) indicated more potential of PSar-IFN (80 pg/mL) than PEG-IFN (136 pg/mL). Moreover, more accumulation of PSar-IFN in tumor site, less exposure in the liver, and lower anti-IFN IgG level in plasma were observed than PEG-IFN in the Sprague–Dawley rat model. Recently, PSar-lipid has been investigated to be used in RNA delivery systems as a PEG alternative [115]. Bi and his colleagues [116] developed a PSar-based lipopolymers to replace PEG-lipid in mRNA lipoplexes. PSar-lipid of different sarcosine units (Mw = 2 k, 5 k) and lipid tail lengths (m = 14, 18) were prepared and used in the mRNA lipoplexes. The results revealed that both the units of sarcosine and lipid tails affect protein expression. PSar shows great potential to replace PEG lipid in mRNA lipid nanoparticles [117] with enhanced protein expression and reduced toxicity compared to LNP formulated with DMG-PEG. Furthermore, researchers also tried to replace PEG in commercial LNP formulations, including SM102 and ALC0315 LNP systems [108]. The results proved PSar’s capability in stabilizing LNP structure and improving RNA delivery efficiency.

#### 4.2.2. Natural Polymers

**Figure 6:**
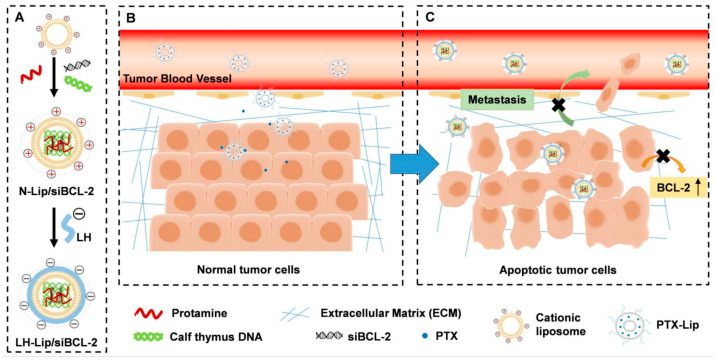
Diagram of chemotherapy priming combined with low-molecular-weight heparin-coated lipid-siRNA complex (LH-Lip/siBCL-2) against pancreatic tumor and metastasis. (**A**) BCL-2 siRNA was encapsulated in cationic liposomes with the help of protamine and calf thymus DNA to form N-Lip/siBCL-2. Then, N-Lip/siBCL-2 was surface-coated with low-molecular-weight heparin to form LH-Lip/siBCL-2. (**B**) A low dose of PTX-Lip was administered as a tumor-priming agent to regulate the tumor microenvironment and promote the delivery of nanodrugs. (**C**) LH-Lip/siBCL-2 was sequentially administrated to inhibit cancer metastasis and downregulate the expression of BCL-2 [119]. Copyright © 2019 Ivyspring International Publisher.

Many natural polymers, such as hyaluronic acid, chitosan, and some saccharide-based polymers, are also considered potential alternatives [105,118]. He Q. et al. developed heparin-coated lipid-siRNA complexes to inhibit cancer metastasis and silence BCL-2 by BCL-2 siRNA (Figure 6) [119]. The tumor was pretreated by paclitaxel-loaded PEGylated liposomes in the study, followed by gene therapy. While it may be a novel model of administration, focusing on the heparin-based siRNA complex highlights heparin and other natural polymers as potential PEG alternatives. Another natural polymer is chitosan, which is the only natural cationic polysaccharide and contains the unit of acetylglucosamine. As chitosan interacts with negatively charged nucleus acid, the formed complex can be used for gene delivery. However, its solubility, targetability, and unpacking ability also limit native chitosan as a gene nanocarrier [120]. Solutions are on the way to modifying the polymer for further usage. At present, there arere still no natural polymers to replace PEG, and many natural polymers need further modification either chemically or physically to meet the requirements.

In summary, advancing both modified PEG lipid derivatives and novel alternative systems necessitates a comprehensive understanding of their underlying mechanisms. Optimization strategies must achieve an equilibrium between therapeutic efficacy and biosafety profiles. Current evidence indicates that branched PEG lipids and polysarcosine (PSar) architectures represent particularly promising candidates. However, further development of more effective strategies remains imperative to fully address the existing challenges in this field.

## 5. Conclusions

LNP technology has established itself as a cutting-edge delivery platform for gene-based therapeutics. Nevertheless, the incorporation of PEG-conjugated lipids as a core excipient in LNP formulations has sparked significant safety debates due to PEG-induced immunogenicity, particularly concerning anti-PEG Abs generation. This immunological response poses critical challenges to the clinical safety profile, therapeutic efficacy, and long-term market potential of nano-biopharmaceuticals. The COVID-19 pandemic notably amplified these concerns, with documented cases of severe hypersensitivity reactions linked to PEGylated formulations. Compounding this issue, epidemiological data reveal an accelerating prevalence of pre-existing anti-PEG Abs in the general population, suggesting widespread PEG exposure. To mitigate risks in LNP-based therapies, rigorous pre-treatment screening and continuous monitoring of circulating APA titers are imperative during PEGylated drug regimens. Concurrently, pharmaceutical innovators are pursuing dual strategies, including structural optimization of PEG lipids through rational design and development of novel PEG-free alternatives. Notably, AI-driven computational approaches are emerging as powerful tools to accelerate the discovery of next-generation lipid alternatives with improved biocompatibility profiles. These developments underscore the necessity for paradigm-shifting solutions to ensure the sustainable advancement of nucleic acid delivery systems.

## Data Availability

Data sharing is not applicable.

## Abbreviations

The following abbreviations are used in this manuscript:

ABC	Accelerating blood clearance
Abs	Antibodies
AMMP	Acoustic membrane microparticle technology
CHAPS	3-[(3-cholamidopropyl)-dimethylammonio]-1-propanesulfonate
CDC	Centre of Disease Control
COVID-19	Coronavirus disease 2019
D	Distance
DSPE	1,2-Distearoyl-sn-glycero-3-phospho-ethanolamine
DXR	Doxorubicin
ELISA	Enzyme-linked immunosorbent assay
GRAS	Generally Recognized As Safe
IC50	half inhibition concentration
LNP	Lipid nanoparticles
mPEG	Methoxy-PEG
MW	Molecular weight
Oligo (ethylene glycol)	EG4
PBS	Phosphate-buffered saline
PEG	Polyethylene glycol
PEGMA	Poly [poly (ethylene glycol) methyl ether methacrylate]
PGs	Poly (glycerols)
PHPMA	Poly (hydroxypropyl methacrylate)
POX	Poly (oxazolines)
PSar	Poly (sarcosine)
PSMA	Prostate-specific membrane antigen
R_F_	Flory radius
SPR	Surface plasmon resonance technology
TD	Thymus-dependent
TFH	Follicular helper T
TI	Thymus-independent
WB	Western blot
